# Crafting the future of bone regeneration: the promise of supramolecular peptide nanofiber hydrogels

**DOI:** 10.3389/fbioe.2025.1514318

**Published:** 2025-03-11

**Authors:** Longbiao Wan, Xiaoyue Yao, Jiali Pan, Ziyang Xiang, Dongjie Fu, Qingsong Ye, Fei Wu

**Affiliations:** ^1^ Department of Orthopedics, Renmin Hospital of Wuhan University, Wuhan, China; ^2^ Department of Ophthalmology, Renmin Hospital of Wuhan University, Wuhan, China; ^3^ Department of Stomatology, Center of Regenerative Medicine, Renmin Hospital of Wuhan University, Wuhan, Hubei, China; ^4^ Sydney Dental School, The University of Sydney, Camperdown, NSW, Australia

**Keywords:** hydrogel composites, bone tissue regeneration, bioactive peptides, scaffold materials, advanced biomaterials

## Abstract

Bone tissue engineering has rapidly emerged as an ideal strategy to replace autologous bone grafts, establishing a comprehensive system centered on biomaterial scaffolds, seeding cells, bioactive factors, and biophysical stimulation, thus paving the way for new horizons in surgical bone regeneration. However, the scarcity of suitable materials poses a significant challenge in replicating the intricate multi-layered structure of natural bone tissue. Supramolecular peptide nanofiber hydrogels (SPNHs) have shown tremendous potential as novel biomaterials due to their excellent biocompatibility, biodegradability, tunable mechanical properties, and multifunctionality. Various supramolecular peptides can assemble into nanofiber hydrogels, while bioactive sequences and factors can be embedded through physical adsorption or covalent binding, endowing the hydrogels with diverse biochemical properties. Finally, this review explored the future challenges and prospects of SPNHs in bone tissue engineering, with the aim of providing insights for further advancements in this field.

## Graphical Abstract

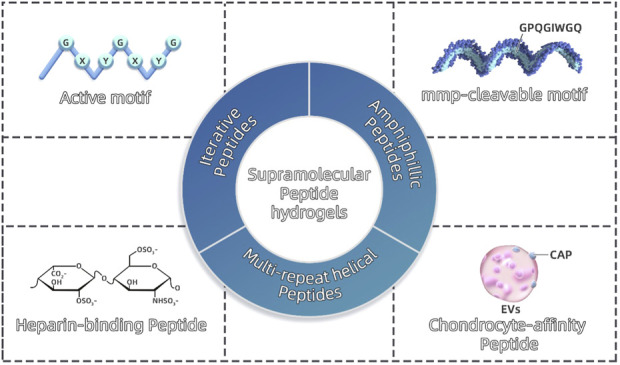

## 1 Introduction

**FIGURE 1:**
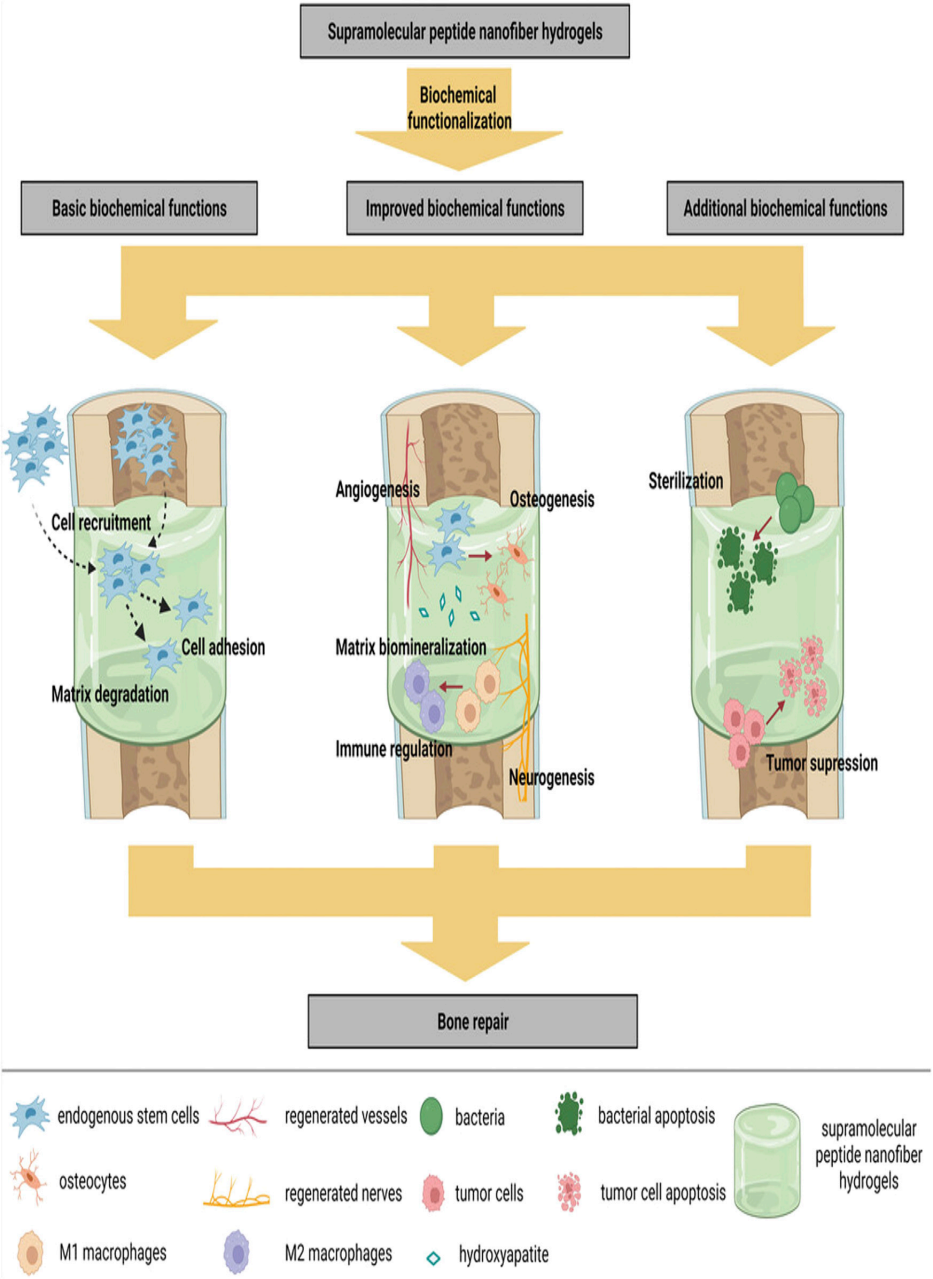
SPNHs are biochemically functionalized to establish a versatile microenvironment. This environment facilitates key processes such as cell adhesion, recruitment, and degradation of the matrix. Additionally, it supports enhanced functions like osteogenesis, angiogenesis, and immune modulation. Moreover, SPNHs offer added benefits, including sterilization and suppression of tumor growth (Zhang J. et al., 2024; Hao et al., 2022). Copyright 2018 WILEY.

The initiation and regulation of tissue repair processes at the site of bone defects are critical for effective fracture healing, involving various tissues, cells, and cytokines from the bone marrow cavity. However, the clinical failure rate of spontaneous bone healing ranges from 5% to 10% (Zura et al., 2016). Currently, autologous bone grafting is still widely regarded as the preferred method in orthopedic interventions of bone regeneration (Zhang J. et al., 2024). It delivers signals that promote osteogenesis, along with osteogenic cells and scaffolds that support bone growth, while also minimizing immune response (Chen et al., 2020), however, the limited availability of autologous bone, along with variable resorption rates, increased morbidity, and the need for additional surgical procedures, resulting in greater patient discomfort and higher costs (Roseti et al., 2017). Allogeneic bone grafting and xenografts are alternative materials; however, their uncontrolled immune responses and infections have emerged as significant concerns that cannot be overlooked (Wang et al., 2024). The bone tissue engineering is built upon four fundamental components: Biomaterial-based scaffolds, stem cells and progenitor cells, active biological factors, and physical signals (Hao et al., 2021; Suamte and Babu, 2024). Among these, hydrogels may represent an ideal scaffold owing to their similar structure to the natural ECM. These materials possess the ability to be classified into natural biomaterials, such as *alginates* (Hernández-González et al., 2020), *collagen* (Nabavi et al., 2020), *chitosan* (Tang et al., 2020) etc., as well as synthetic biomaterials (Dai et al., 2022). Among synthetic biomaterials, peptide-based hydrogels exhibit excellent biocompatibility, biodegradability, high purity, ease of functionalization, and tunable mechanical properties. These emerging tissue engineering biomaterials can form nanofibers and subsequently create a nanonetwork under specific conditions (such as *pH*, *temperature*, and *shear stress*), resulting in Supramolecular Peptide Nanofiber Hydrogels (SPNH). Currently, SPNHs are applied across various medical fields. In this work, we examined the biocheclude basic roles such as cell adhesion, recruitment, and matrix degradation, as well as enhanced roles like osteogenesis, neuroangiogenesis, and immunomodulation, along with additional functionalities like sterilization and tumor suppression (Figure 1). Despite significant progress in SPNHs, key gaps remain in understanding their long-term behavior *in vivo*, including degradation rates, stability, and interactions with the physiological environment. The aims of this review are to examine the fundamental, improved, and additional biochemical functions of SPNHs in bone regeneration, identify gaps in current research, and suggest future directions.

## 2 Bone-related microenvironment

Bone can be classified targeting the compact and trabecular bone regions. Compact bone consists of densely arranged bone units, which are made up of Haversian systems containing vascular and neural tissues. Surrounding these units are concentric lamellae of Collagen fibers are supported by hydroxyapatite (HAP). In addition, non-collagenous proteins, such as laminin and fibronectin, also contribute to this reinforcement (Linder et al., 2020). Osteocytes exist within lacunae of bone units and are immersed in a matrix that contains a high concentration of proteoglycans as well as bioactive factors (Sui et al., 2023). SPNHs, resembling ECM, possess high water content and multilayered nanostructures. However, to more closely mimic the biological functions of ECM, current approaches focus on integrating bioactive motifs, for example, cell adhesion peptides (CAPs), as well as biologically active molecules like TGF-β, which are crucial for various cellular processes (Liu et al., 2020; Eskandari et al., 2017; Wan et al., 2025). In comparison with currently utilized biocompatible materials, SPNHs can be endowed with a variety of biochemical works via integrating active biological sites as well as adsorbing biological activators, significantly accelerating the repair of bone defects (Hao et al., 2022). A summary of multiple biofunctional motifs and agents is provided (Table 1).

**TABLE 1:** Biofunctional elements and factors in bone repair applications.

Motifs or factors	Fundamental purpose	Additional functions	Citation
RGD,RGDS, PRGDSGYRGDS(PRG), DGRGDSVAYG (DGR)	Cell adhesion (bioactive motifs)	Osteogenesis, neurogenesis, angiogenesis	Eskandari et al. (2017), Wan et al. (2025), Huettner et al. (2018)
PHSRN	Cell adhesion (bioactive motifs)	cell proliferation–	Matsugami et al. (2021)
RPKPQQFFGLM (substance P, SP)	Cell recruitment (bioactive motifs)	Osteogenesis, angiogenesis	Abioye et al. (2024), Restu et al. (2020), Lu et al. (2018)
Interleukin-8 (IL-8)	Cell recruitment (bioactive factors)	–	Zhang et al. (2024b)
PTGXKV	Matrix degradation (bioactive motifs)	Motif delivery	Lin et al. (2019)
GPQGIWGQ		Motif delivery	Comazzetto et al. (2021)
Bone morphogenetic proteins (BMPs)	Osteogenesis (bioactive factors)	Angiogenesis	Tavakol et al. (2019)
Extracellular vesicles (EVs)		Angiogenesis	Gentile et al. (2017)
LRKKLGKA	Osteogenesis (bioactive motifs)	By utilizing heparan sulfate to mediate interaction with BMP-2, osteogenesis is enhanced	Shi et al. (2019)
SpSVPTNSPVNSKIPKACCVPTELSAI (BMP-2-mimetic peptide)	Osteogenesis	osteoblast differentiation、Chondrogenesis and repair	Tavakol et al. (2019)
RKKNPNCRRH (BMP-4-mimetic peptide)	Osteogenesis、Chondrogenesis	Promotes osteoblast and chondrocyte differentiation、Enhances bone defect repair	Tavakol et al. (2019)
GQGFSYPYKAVFSTQ (BMP-7-mimetic peptide)	Osteogenesis	Repair kidney tissue、Anti-fibrotic effect	Tavakol et al. (2019)
CGGKVGKACCVPTKLSPISVLYK (BMP-9-mimetic peptide)	Osteogenesis、Chondrogenesis	Angiogenesis、Regulation of bone metabolism and vascular-bone interactions	Tavakol et al. (2019)
DGEA		Selective adhesion for osteoblasts by integrin *α*2*β*1	Huang et al. (2019), Rabenstein (2002)
GFOGER		Selective adhesion for osteoblasts by integrin *α*2*β*1	Lee et al. (2013)
GTPGPQGIAGQRGVV		Selective adhesion for osteoblasts by integrin *α*2*β*1	Lee et al. (2017)
KRSR		Selective adhesion for osteoblasts by cell-membrane heparin sulfate proteoglycans	Amirahmadi et al. (2023)
VEGF-related factors	Vascularization (bioactive factors)	Osteogenesis	Wan et al. (2024)
Fibroblast growth factor 2 (FGF-2)		Osteogenesis	Keshtkar et al. (2018)
Insulin-like growth factors (IGFs)		Osteogenesis	Shao et al. (2018)
Nerve growth factor (NGF)	Neurogenesis (bioactive factors)	Osteogenesis	Wang et al. (2021b), Lin et al. (2012), Hosseinkhani et al. (2006)
Brain-derived neurotrophic factor (BDNF)		Osteogenesis	Wang et al. (2021b), Lin et al. (2012), Hosseinkhani et al. (2006)
RGIDKRHWNSQ (BDNF-mimetic peptide)	Neurogenesis (bioactive motifs)	–	Bakshi et al. (2021)
Cyclic RKKADP (BDNF-mimetic peptide)	Promotes neuronal survival and differentiation	Promotes nerve repair and regeneration、Promotes synaptic plasticity	Bakshi et al. (2021)
EVYVVAENQQGKSKA (FGL)	Cell proliferation and differentiation	Antifibrosis、Promotes tissue repair and regeneration	Sun et al. (2020)
SIDRVEPYSSTAQ (FRM)	fibroblast recruitment	Angiogenesis、Antifibrosis	Liu et al. (2021)
IKVAV		Laminin-mediated cell adhesion protein	Liu et al. (2018)
YIGSR		Laminin-mediated cell adhesion protein angiogenesis	Liu et al. (2018)
RNIAEIIKDI		Laminin-mediated cell adhesion protein	Liu et al. (2018)
IL-4	Immune regulation	anti-inflammatory	Lu et al. (2019)
Antimicrobial peptides (AMPs)	Sterilization		Zou et al. (2014)

## 3 Fundamental biochemical functions

### 3.1 Cell adhesion

Cell adhesion is a fundamental function of biomaterials, typically mediated by CAPs, which interact with designated cell receptors membrane, such as integrins and fibronectin-binding proteins. Several CAPs target integrins, with the fibronectin-derived RGD peptide being widely utilized due to its effectiveness in promoting cell attachment (Huettner et al., 2018). RGD can bind to multiple integrins, thereby activating additional processes like osteogenesis, angiogenesis, and neurogenesis. RGD peptides coupled with their products, for instance *RGDS*, *PRGDSGYRGDS* (PRG), and *DGRGDSVAYG* (DGR), Undergo tailoring into different types of supramolecular peptides to create bioactive hydrophobic hydrogels (Hao et al., 2022; Matsugami et al., 2021; Liu et al., 2012; Luo et al., 2019). A peptide inspired by fibronectin, designed to enhance cell attachment Pro-His-Ser-Arg-Asn (PHSRN)PHSAA recent findings indicate that synergistically improve cell attachment and cell multiplication when combined with RGD in supramolecular peptides (Aye et al., 2018).

The mechanical features of SPNHs play a crucial role in promoting cell attachment, spreading, along with differentiation. Such stiffness and elasticity of SPNHs directly affect cellular behavior, as materials that are too soft or rigid may impair cell attachment and function (Hao et al., 2022; Wang S. et al., 2021). By adjusting the mechanical properties to align with the target tissue, such as *bone*, it is possible to enhance integrin engagement, which in turn promotes stronger cell adhesion and proliferation. When combined with biochemical signals, such as the RGD sequence in CAPs, this mechanical stimulation creates a synergistic effect that promotes tissue regeneration (Abioye et al., 2024). Balancing both mechanical and biochemical properties makes SPNHs an ideal platform for bone repair and regeneration.

### 3.2 Cell recruitment

**FIGURE 2:**
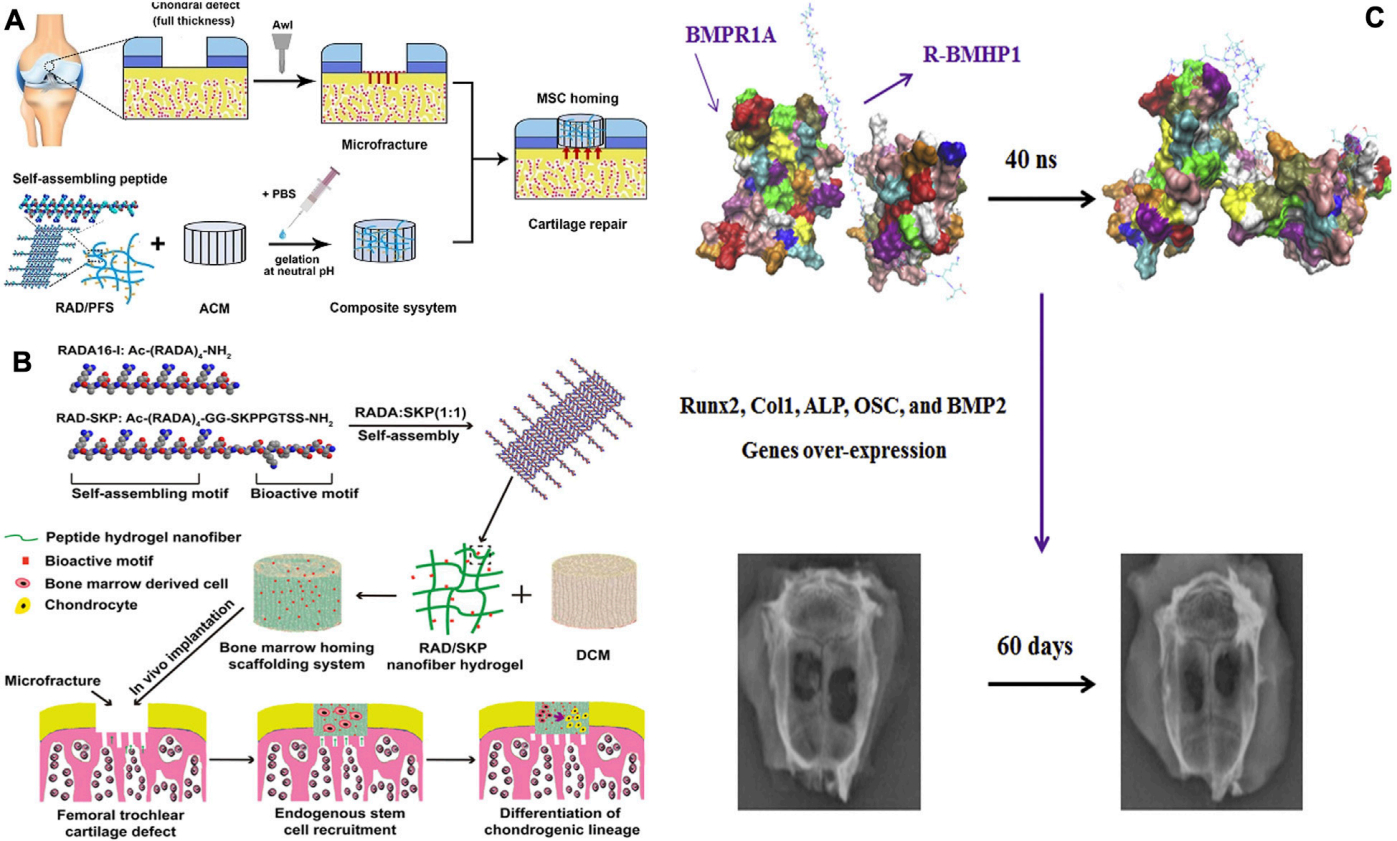
**(A)** A hybrid hydrogel structure was created by integrating a directional acellular cartilage matrix (ACM) and a self-assembling peptide (SAP) conjugated having a bone marrow homing peptide (BMHP). It was assumed that the scaffold’s role in attracting intrinsic Msc could foster the regeneration of cartilage tissue (Comazzetto et al., 2021). **(B)** A promising scaffolding strategy, designed to repair osteochondral defects in rabbits, combines a scaffold derived from decellularized cartilage matrix (DCM) utilizing a peptide hydrogel that assembles on its own. The hydrogel incorporates Ac-(RADA)4-CONH2 and Ac-(RADA)4GGSKPPGTSS-CONH2 (RAD/SKP) to enhance the regenerative potential (Lu et al., 2018). **(C)** A model obtained through binding simulation was employed as the starting conformation for molecular dynamics (MD) simulations. The R-BMHP1 was represented as an orange strand, while the receptor was shown with a gray outline; nevertheless, R-BMHP1 occupied the binding regions of the receptor. The interaction strength of various amino acids points to the significance of specific arginine residues (ARG1, ARG5, ARG9, and ARG13) in the R-BMHP1 nanofiber strand, along with LYS24 from the BMHP1 peptide, in interacting with the BMPR1A receptor. Subsequent to the charge-driven interaction, osteogenesis-related genes were significantly elevated, facilitating the repair of bone tissue (Tavakol et al., 2019). Copyright 2019, Wiley-VCH.

Cell recruitment involves the ability of biomaterials to attract endogenous repair cells from niches like the bone marrow, promoting their migration into the material. This reduces reliance on exogenous seed cells, thereby lowering costs and enhancing the body’s natural healing response. SPNHs offer a porous, ECM-mimetic microenvironment that promotes cell recruitment, a process that can be further augmented by integrating bioactive elements. Whereas these substances mimic such porous architecture of the extracellular matrix (ECM) to promote endogenous cell migration, current limitations lie in the precise control of bioactive motif release kinetics. Future designs could focus on spatiotemporal modulation of signaling molecules to optimize recruitment efficiency. Bone marrow homing peptides (BMHPs), containing high levels of K, P, F, S, along with T, can be identified through phage display as effective in promoting MSC migration. Incorporating BMHPs into SPNHs can optimize regenerative outcomes by leveraging the body’s intrinsic repair mechanism (Restu et al., 2020). Lu et al. (2018) developed composite nanoscaffolds Via the integration of decellularized cartilage matrix (DCM) with RADA 16 water-based gel otherwise RADA 16/RADA 16-GG-PFSSTKT hydrogel. Their findings demonstrated that both RADA 16/RADA 16-GG-PFSSTKT and RADA 16/RADA 16-GG-SKPPGTSS hydrogels effectively promoted subchondral bone regeneration within the DCM. Although these studies primarily focused on osteogenic differentiation and gene expression, they also highlighted the potential of these hydrogels for directing MSCs to the injury area, as evidenced by increased cellular infiltration in the defect area. Additionally, the concurrent application of BMHP1 with crosslinked RADA 16 significantly amplified alkaline phosphatase levels and upregulated expression of bone-related genes in MSCs (Figure 2). Existed experimental results further confirmed that RADA 16/RADA 16-GG-PFSSTKT scaffold accelerates regeneration in rodent skull lesions (Cui et al., 2022). Additionally, Substance P (SP), a neuropeptide with the sequence RPKPQQFFGLM, plays a role in neurological functions known for its ability to recruit MSCs (Zhang K. et al., 2024). Upon subcutaneously implanting poly (lactic acid) (PLA) scaffolds containing KLD 12-/KLD 12-SP hydrogels in nude mice, it was observed that the PLA hydrogels exhibited the highest homing activity, recruiting the most labeled MSCs. Furthermore, several active biomolecules, like *stromal cell-derived factor 1β* (SDF-1β) (Raftery et al., 2024)along with *interleukin-8* (IL-8) (Lin et al., 2019))demonstrated cell migration properties and can be physically encapsulated within SPNHs for enhanced bone regeneration (Table 1).

### 3.3 Matrix degradation

**FIGURE 3:**
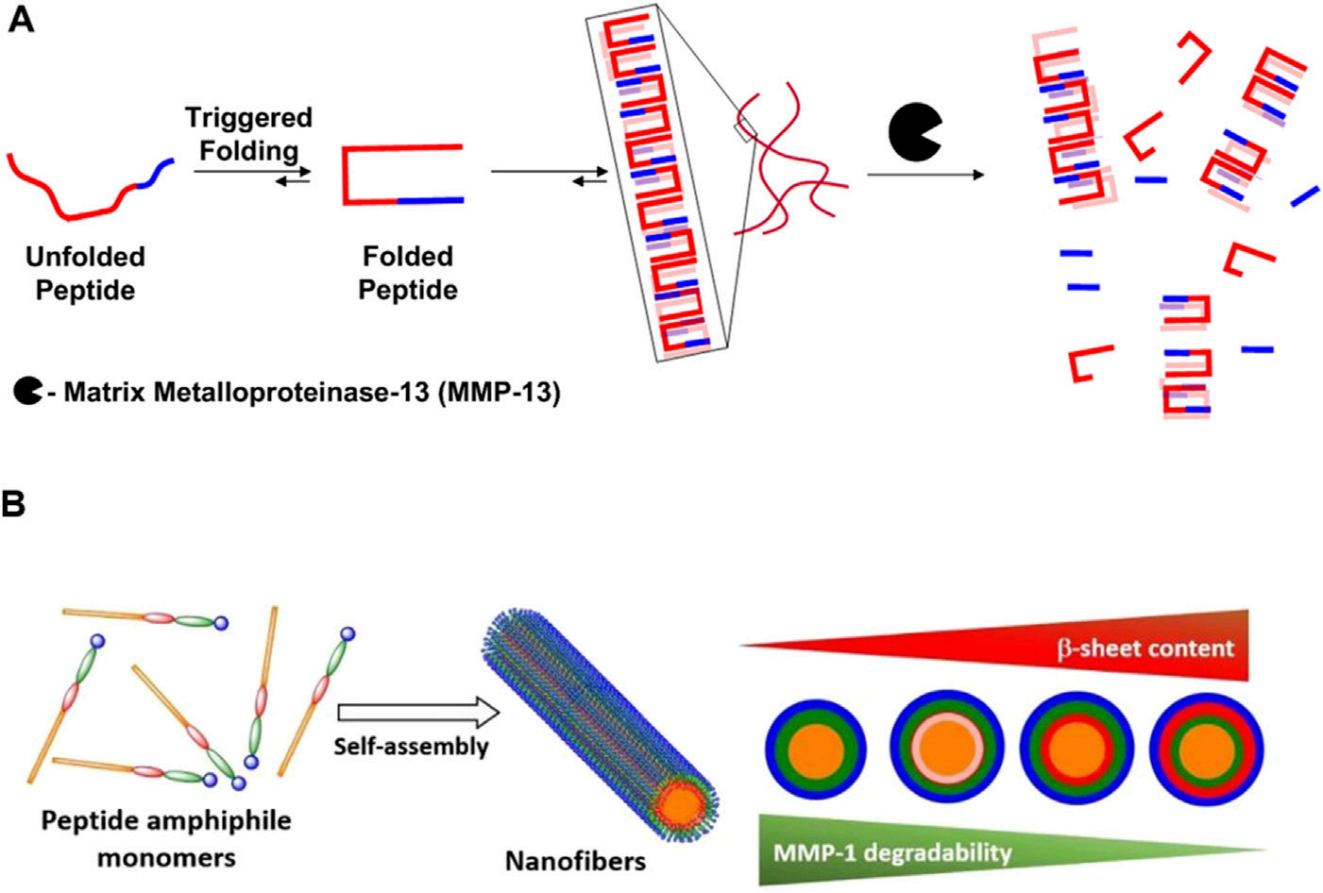
**(A)** The environment induces folding and self-organization, resulting in the development of a gel-like structure. The following biological disintegration of β-hairpin hydrogels (Giano et al., 2011). **(B)** A relationship within the MMP-1 breakdown effectiveness, coupled with the β-sheet proportion in the self-assembled PA nanofibers., this MMP-1 cleavage activity was markedly lowered in PA nanofibers that displayed enhanced β-sheet formation (Shi et al., 2019). Copyright 2022 Elsevier.

Hydrogel matrices should provide niches that facilitate the infiltration of endogenous cells, ensuring that the scaffold can effectively degrade during the bone defect repair process. Moreover, the speed of decomposition in the matrix should be carefully balanced in accordance with tissue development speed regeneration into optimize healing outcomes (Kou et al., 2021). One strategy involves using diverse sequences that exhibit varying response rates to matrix metalloproteinases (MMPs), potentially resulting in distinct physical performance of the SPNHs. Giano et al. (2011) implemented various MMP-13-cleavable sequences into β-hairpin peptides following the PTGXKV pattern, substituting different residues at the X position: phenylalanine for Decapeptide 1 (DP1), leucine to Decapeptide 2 (DP2), isoleucine to Decapeptide 3 (DP3), along with alanine in Decapeptide 4 (DP4) (Hao et al., 2022). This data indicated a degradation rate order of the different hairpin structure peptides in the following manner: DP 1 > DP 2 > DP 3 > DP 4 (Giano et al., 2011). A possible cause of this is that DP one exhibits the lowest mechanical properties, potentially facilitating MMP-13’s penetration and degradation of the matrix. Another approach involves linking MMP-cleavable sequences through distinct spacer regions characterized by unique secondary structures. The MMP-1-cleavable sequence (GPQGIWGQ) was connected to hydrophobic alkyl chains via different spacer regions: Peptide Assembly 1(PA1), Peptide Assembly 3(PA3), and Peptide Assembly 4(PA4) (well-known folding sequences), and Peptide Assembly 2(PA2) (exhibiting 20% helical structure). Results indicated degradation did not appear in PA three along with PA 4 nano-scale filaments over 70 h, while PA one nanofibers exhibited detectable degradation (0.21%) within 24 h (Shi et al., 2019). In the case of PA 2, a degradation rate of 3.22% was observed after 24 h. These results suggest that the secondary structure of the spacer can influence degradability by affecting the availability of the active sites (Shi et al., 2019). (Figure 3) This expands the possibilities for selecting SPNHs.

## 4 Improved biochemical functions

### 4.1 Osteogenesis

**FIGURE 4:**
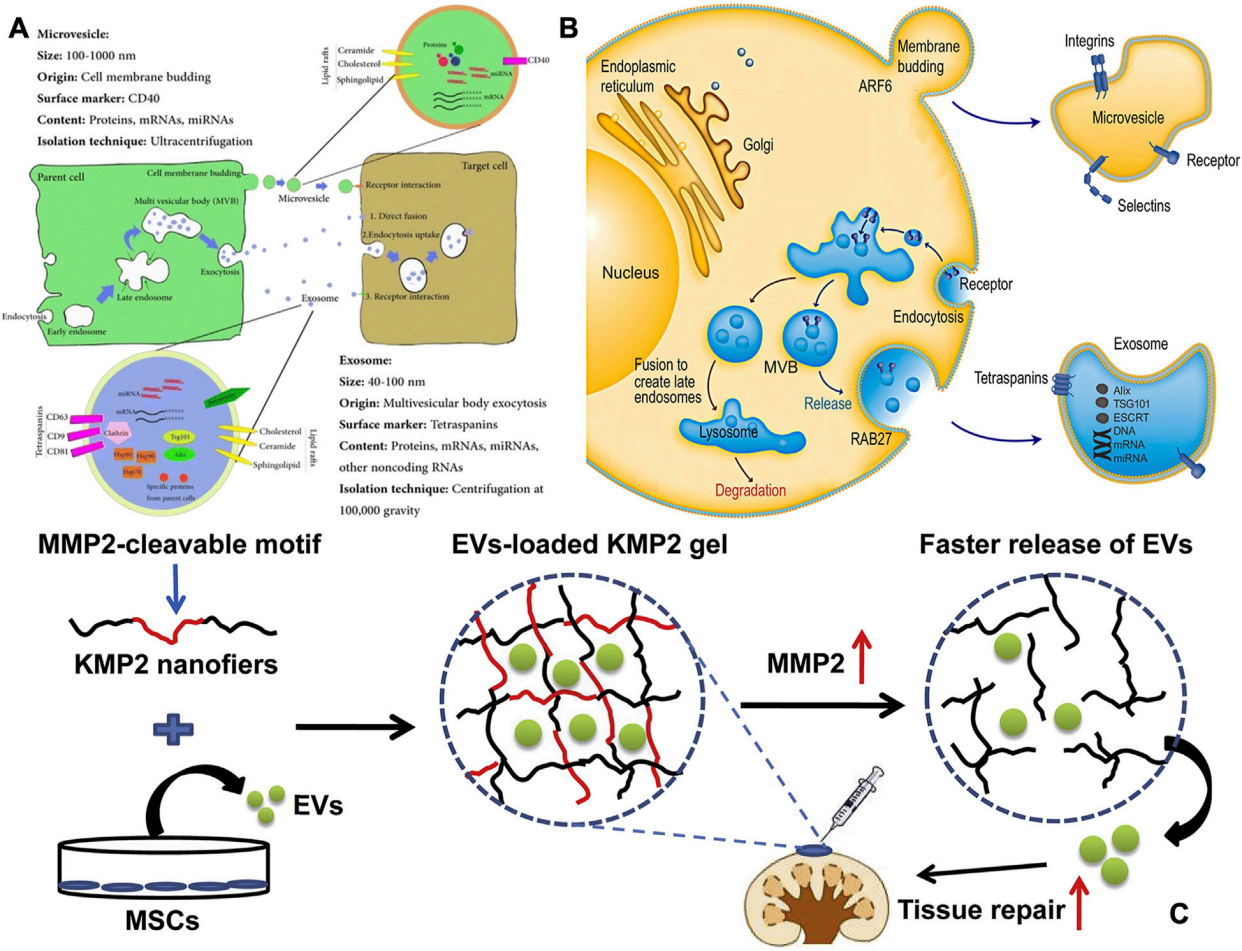
**(A)** The source, makeup, and intersomatic interactions of EVs. The terms Hsp (heat shock protein), MVB (multivesicular body), and Tsg 101 (tumor susceptibility gene 101) are used to denote specific cellular components (Keshtkar et al., 2018). **(B)** Intracellular mechanisms of extracellular vesicle formation and release. Extracellular vesicles are secreted by cells via one of which the external protrusion of the plasma bilayer (microvesicle pathway) or the internal folding of the endosomal bilayer (Exosomal route). Exosomes are membrane-bound vesicles formed through endocytosis. After the plasma bilayer folds inward to generate the early endosome, exosomes are produced as internal vesicles by additional inward folding of the boundary bilayer of the endosome, which is now identified as the multivesicular body (MVB). Ultimately, exosomes are released when the MVB merges with the plasma bilayer. Various cellular machineries play a role in controlling cargo packaging and the release of exosomes (Shao et al., 2018). Copyright 2011 abcam. **(C)** To facilitate the local delivery of MSC-EVs, a matrix metalloproteinase-2 (MMP2)-responsive self-assembling peptide hydrogel (KMP2) was utilized. It demonstrated enhanced kidney function by decreasing cell death in tubular cells, expression of Inflammatory signaling molecules, and invasionof macrophages (Firoozi et al., 2020). Copyright 2011 abcam.

In bone tissue engineering, SPNHs can enhance osteogenesis by incorporating bone-inducing factors or peptides. Among the most widely used bioactive proteins are recombinant BMPs, including BMP-2, BMP-3, BMP-4, BMP-6, BMP-7, BMP-9, and BMP-12, which exhibit osteoinductive activity. Studies have shown that when BMPs are encapsulated within peptide amphiphiles (PAs), they promote healing in rabbit cranial defects (Fichman and Schneider, 2020). However, BMPs face challenges such as purification difficulties, high costs, supraphysiological dosing, and rapid release rates. To address these issues, enhancing the affinity between SPNHs and bioactive factors is an effective strategy. Heparan sulfate (HS), a glycosaminoglycan component of the ECM, is able to non-covalently bind to bioactive proteins, improving signal transduction in osteogenesis by stabilizing receptors and protecting proteins from hydrolysis, ultimately enhancing bone formation and mineralization (Huang et al., 2019; Rabenstein, 2002). This interaction can enhance signal transduction, stabilize receptors, and protect proteins from hydrolysis (Figure 4). Lee et al. (2013) developed the functionalized PA with heparin-binding peptides (LRKKLGKA) engineered to mimic these interactions, demonstrating collagen-based hydrogel composites containing BMP-2 along with HS reduce BMP-2 dosage by tenfold while promoting bone regeneration compared to collagen/BMP-2 composites. However, animal-sourced HS in clinical settings is constrained by poor bioavailability and potential side effects. To overcome this, sulfated monosaccharides have been employed to simulate natural polysaccharides and conjugated to PA (Lee et al., 2017). ECM-derived peptides, such as *RGD*, *DGEA* (Amirahmadi et al., 2023), *GFOGER* (Ha et al., 2023), *P-15* (GTPGPQGIAGQRGVV) (Atieh et al., 2021),and *KRSR* (lysine-arginine-serine-arginine) (Gentile et al., 2017), can also selectively bind to osteoblasts, promoting biomineralization. Additionally, extracellular vesicles (EVs), rich in bioactive components, have emerged as promising materials for promoting osteogenesis and angiogenesis (Dee et al., 1998; Wang et al., 2022). Nevertheless, their clinical utility in bone tissue engineering is constrained by rapid degradation and systemic clearance. The integration of CAPs into peptide hydrogels significantly strengthens EV-matrix interactions, thereby optimizing their therapeutic potential for bone repair applications (Firoozi et al., 2020; Wan et al., 2024). Clinically, SPNHs serve as excellent drug carriers, holding promise for the delivery of osteoinductive drugs, thus emerging as potential materials for bone tissue engineering.

### 4.2 Neuroangiogenesis

Taking into account that bone has a dense vascular network and innervated structure, both blood vessels and nerves play crucial roles in bone metabolism, remodeling, cellular function, and nutrient exchange. This intricate network not only supports the mechanical integrity of bone but also regulates various biological processes essential for maintaining bone health and facilitating repair mechanisms (Fan et al., 2014). When passive diffusion of oxygen and nutrients is insufficient to promote bone regeneration, angiogenesis and neurogenesis become crucial for the repair of injured bone. New blood vessel formation is critical for supplying nutrients and oxygen, while nerve regeneration supports cellular signaling and metabolic regulation necessary for effective healing (Wang B. et al., 2021). Therefore, incorporating angiogenic signals into biomaterials enhances bone regeneration. Various bioactive proteins play pivotal roles in angiogenesis, including VEGF (Lin et al., 2012), bFGF (Hosseinkhani et al., 2006) along with IGFs, all critical for cellular growth and repair (Kang et al., 2012). VEGF is widely recognized for its role in inducing the formation of new blood vessels (Hao et al., 2022). To illustrate, linking VEGF to BMP-2 and tangibly encapsulating it in PA hydrogels loaded into collagen demonstrated enhanced bone regeneration in a rat model of extensive skull damage. This underscores the significant influence of angiogenesis in bone regeneration (Bakshi et al., 2021).

NGF and BDNF are vital bioactive molecules involved in the formation and regeneration of neural tissue, both of which influence bone formation either directly or indirectly (Sun et al., 2020; Liu et al., 2021; Liu et al., 2018). The combination of neurotrophic molecules and osteogenic agents within SPNHs has the potential to stimulate bone repair, as these hydrogels are widely explored for use in neural along with neurotissue engineering (Koss et al., 2016). Neurodevelopmental sequences extracted through neurotrophic factors comprise peptides that mimic BDNF (e.g., RGIDKRHWNSQ, cyclic RKKADP) (Lu et al., 2019) coupled with emanating from neural cell anchoring factors sequences (e.g., EVYVVAENQQGKSKA (Wang et al., 2015) and SIDRVEPYSSTAQ (Zou et al., 2014)), which are designed to promote neurogenesis. Neurogenic components obtained in the native extracellular matrix, such as *Emanating from laminin sequences* (e.g., IKVAV, YIGSR, and RNIAEIIKDI), are applied in the field of NTE. Notably, both IKVAV and YIGSR have proven capable of stimulating angiogenesis as well (Jain and Roy, 2020).

### 4.3 Immunomodulation

The importance of this immune system in regulating osteogenesis is crucial, particularly the involvement of several components of the immune response, like neutrophils, macrophages, and T lymphocytes. Macrophages, specifically, play a significant role; initially exhibiting an M1 phenotype that facilitates the uptake of apoptotic cells coupled with pathogens while promoting inflammation, they subsequently transition to an anti-inflammatory M2 phenotype, which stimulates osteogenesis (Jiang et al., 2021). Current research has prioritized dual modulation of macrophage phenotypes by suppressing M1 polarization and enhancing M2 activation. To achieve this, interleukin-4 (IL-4)—a potent M2-polarizing cytokine—was covalently conjugated with BMP-2 conjugated with graphene oxide (GO) to establish a controlled release platform. This IL-4/BMP-2 functionalized GO system was subsequently encapsulated within carboxymethyl chitosan/polyethylene glycol diacrylate (CMC/PEGDA) hybrid hydrogels. Experimental studies *ex vivo* showed that such dual-factor hydrogel synergistically encouraged M2 macrophage activation and bone regeneration, while *in vivo* evaluations demonstrated significant inflammation suppression coupled with enhanced bone formation (Zou et al., 2021). Additionally, certain immunomodulators can be incorporated as drug carriers into SPNHs for applications in bone tissue engineering.

## 5 Additional biochemical functions

### 5.1 Sterilisation

Acute and chronic bone infections present significant treatment challenges due to bacterial colonization and acidic microenvironments (Fang et al., 2021). Osteomyelitis, typically caused by infections leading resulting in bone tissue response otherwise bone marrow, is typically managed with completely removing the affected tissue, followed by the implantation of antimicrobial materials. SPNHs serve as a perfect support system functioning in two ways, with scaffolding as one along with controlled release, providing both antibacterial and osteogenic properties. Yang et al. (2018) included positively charged antimicrobial peptides (Amps) within RADA 16 hydrogels, achieving controlled release of Amps in a rabbit osteomyelitis model. This approach effectively inhibited the multiplication of *S. aureus* as well as promoted bone repair. Additionally, encapsulating ciprofloxacin within RADA 16/calcium phosphate cement scaffolds demonstrated significant efficacy in preventing postoperative infections (Li et al., 2021).

### 5.2 Tumour suppression

Scaffolds with combined capabilities of tumor inhibition and bone regeneration offer a potential treatment for bone abnormalities resulting from tumors. Antitumor agents with osteogenic properties, such as *metformin*—a diabetes medication that inhibits tumors and promotes bone formation—were successfully incorporated into scaffolds, inducing multiple beneficial functions, including both antitumor effects and enhanced bone regeneration (Tan et al., 2021). Currently, there are few studies investigating SPNHs as controlled release scaffolds. Conjugating antitumor agents to SPNHs presents a promising strategy for enhancing their therapeutic efficacy in bone regeneration.

## 6 Discussion

Bone regeneration depends on the complex interplay of multiple processes, where angiogenesis and neurogenesis are key to successful healing. SPNHs hold great promise in this context, offering a versatile matrix that can simultaneously support both vascular and neural regeneration, critical for bone repair. When passive diffusion of oxygen and nutrients is inadequate, angiogenesis becomes essential to supply the necessary resources for healing. SPNHs have been shown to enhance blood vessel formation by incorporating pro-angiogenic peptides, such as VEGF and BMP-2, which stimulate endothelial cell migration and capillary growth within the scaffold. This vascularization is crucial for bone regeneration, as it facilitates nutrient delivery and regulates osteogenesis. Despite their promising potential, there are still several challenges in the development of SPNHs. The fabrication process can be complex, resulting in variations in mechanical properties and biological performance. Controlling degradation rates is also critical to prevent premature breakdown of the scaffold. Additionally, issues related to cost-effectiveness and scalability for large-scale production remain significant hurdles. While the mechanical properties of SPNHs can be adjusted, they may still fall short of matching the strength and elasticity of natural bone, especially in applications that require high load-bearing capacity.

In addition, neurogenesis is crucial for bone reconstruction through influencing osteoblast function and modulating inflammation. SPNHs can be designed to release neurotrophic factors like NGF and BDNF, promoting nerve growth and enhancing the overall regenerative process. The dual role of SPNHs in supporting both angiogenesis and neurogenesis offers a unique advantage in addressing the complex needs of bone healing. By delivering multiple bioactive factors in a controlled manner, SPNHs create an integrated microenvironment that accelerates bone restoration process.

Whereas other strategies are also adopted to facilitate bone renewal, like hydrogels mimicking the bone ECM, the incorporation of bioactive elements into scaffolds, and the use of EVs, SPNHs offer distinct advantages. Hydrogels mimicking the hierarchical structure of bone ECM aim to replicate the natural architecture of bone but may not offer the same level of functional versatility or tunable mechanical properties as SPNHs. Bioactive element-doped scaffolds, such as those incorporating *strontium* or *calcium*, can enhance osteogenesis; however, they lack the ability to dynamically release multiple bioactive factors, an ability that SPNHs possess. Furthermore, while EVs show promise in bone regeneration, their rapid clearance and short half-life limit their long-term effectiveness. In contrast, SPNHs offer a sustained release system with controllable degradation rates, providing prolonged therapeutic effects. Therefore, the ability of SPNHs to deliver a combination of bioactive signals, along with their customizable mechanical properties, places them ahead of other approaches in terms of promoting bone regeneration.

## 7 Conclusion

In summary, SPNHs are an innovative biomaterial that holds great promise for advancing tissue engineering, particularly in bone regeneration. Their unique properties allow for multifaceted applications, which can significantly improve skeletal healing outcomes. While extensive animal studies have validated their efficacy, the next phase of research must pivot towards clinical applications to establish robust evidence of their safety and effectiveness in human subjects.

## 8 Future perspectives

Future research should prioritize clinical trials to validate the effectiveness of SPNHs in diverse settings. Exploring their potentials to surpass traditional natural and synthetic hydrogels could pave the way for broader clinical adoption. Additionally, investigating the long-term performance of SPNHs *in vivo* will be crucial for understanding their durability and integration within host tissues. Emphasizing personalized approaches in biomaterial design could further enhance the therapeutic potential of SPNHs, positioning them as a cornerstone in regenerative medicine.
